# Sex differences in the human brain related to visual motion perception

**DOI:** 10.1186/s13293-024-00668-2

**Published:** 2024-11-11

**Authors:** Dong-Yu Liu, Ming Li, Juan Yu, Yuan Gao, Xiaotong Zhang, Dewen Hu, Georg Northoff, Xue Mei Song, Junming Zhu

**Affiliations:** 1 Department of Neurosurgery of the Second Affiliated Hospital, Zhejiang University School of Medicine, Hangzhou, 310029 China; 2 College of Intelligence Science and Technology, National University of Defense Technology, Changsha, 410073 China; 3 Key Laboratory of Biomedical Engineering of Ministry of Education, Interdisciplinary Institute of Neuroscience and Technology, College of Biomedical Engineering and Instrument Science, Zhejiang University, Hangzhou, 310027 China; 4 MOE Frontier Science Center for Brain Science & Brain-Machine Integration, Zhejiang University, Hangzhou, 311121 China; 5 College of Electrical Engineering, Zhejiang University, Hangzhou, 310027 China; 6 University of Ottawa Institute of Mental Health Research, University of Ottawa, Ottawa, ON K1Z 7K4 Canada

**Keywords:** Motion perception, Human MT complex, Gray matter volume, Amplitude of low-frequency fluctuations, Sex differences

## Abstract

**Background:**

Previous studies have found that the temporal duration required for males to perceive visual motion direction is significantly shorter than that for females. However, the neural correlates of such shortened duration perception remain yet unclear. Given that motion perception is primarily associated with the neural activity of the middle temporal visual complex (MT+), we here test the novel hypothesis that the neural mechanism of these behavioral sex differences is mainly related to the MT+ region.

**Methods:**

We utilized ultra-high field (UHF) MRI to investigate sex differences in the MT+ brain region. A total of 95 subjects (48 females) participated in two separate studies. Cohort 1, consisting of 33 subjects (16 females), completed task-fMRI (drafting grating stimuli) experiment. Cohort 2, comprising 62 subjects (32 females), engaged in a psychophysical experiment measuring motion perception along different temporal thresholds as well as conducting structural and functional MRI scanning of MT+.

**Results:**

Our findings show pronounced sex differences in major brain parameters within the left MT+ (but not the right MT+, i.e., laterality). In particular, males demonstrate (i) larger gray matter volume (GMV) and higher brain’s spontaneous activity at the fastest infra-slow frequency band in the left MT+; and (ii) stronger functional connectivity between the left MT+ and the left centromedial amygdala (CM). Meanwhile, both female and male participants exhibited comparable correlations between motion perception ability and the multimodal imaging indexes of the MT+ region, i.e., larger GMV, higher brain’s spontaneous activity, and faster motion discrimination.

**Conclusions:**

Our findings reveal sex differences of imaging indicators of structure and function in the MT+ region, which also relate to the temporal threshold of motion discrimination. Overall, these results show how behavioral sex differences in visual motion perception are generated, and advocate considering sex as a crucial biological variable in both human brain and behavioral research.

**Supplementary Information:**

The online version contains supplementary material available at 10.1186/s13293-024-00668-2.

## Plain language summary

There are sex differences in visuospatial abilities between males and females, including the visual perception of motion information. However, the neural mechanism of these sex differences in motion perception remains yet unclear. To explore this question, we employed the joint task probing motion perception and ultra-high field (UHF) MRI. We found that the motion discrimination was faster in males compared to females. The sex differences were also prominent in major brain parameters in the MT+ region (the function brain regions of motion perception). Males demonstrate (i) larger gray matter volume (GMV) and higher brain’s spontaneous activity in the left MT+ (but not right MT+, i.e., laterality); (ii) stronger functional connectivity between the left MT+ and the left centromedial amygdala (CM). Additionally, we observed that female and male participants exhibited comparable correlations between motion perception ability and the multimodal imaging indexes in the MT+ region, i.e., the larger GMV, the higher brain’s spontaneous activity, and the faster motion discrimination. These results suggest that the sex differences in the structure and function of the MT+ region are the neural mechanism underlying the behavioral-level sex differences in motion perception. We demonstrate sex differences in the healthy human MT+ of the brain, possibly leading to sex differences in visual perception. This strongly support the consideration of sex as a crucial biological variable in both human brain and behavioral research.

**Supplementary Information:**

The online version contains supplementary material available at 10.1186/s13293-024-00668-2.

## Highlights

- In the left MT+ region, males exhibit larger GMV and higher spontaneous activity at the fastest infra slow frequency band compared to females.
- The functional connection between left MT+ and left centromedial amygdala in males is stronger than that in females.
- Female and male participants demonstrated comparable correlations between motion perception ability and the multimodal imaging indexes in the MT+ region (the function brain region of motion perception), i.e., the larger GMV, the higher spontaneous activity in MT+, and the faster motion discrimination.
- The relationship of motion discrimination and left MT+ GMV is indirect-only and mediated by the spontaneous activity of left MT+ in the fastest infra-slow frequency band.

**Supplementary Information:**

The online version contains supplementary material available at 10.1186/s13293-024-00668-2.

## Introduction

Sex differences have always been a behavioral issue of concern [1, 2], but our knowledge about their relevance to human brain is sparse [3, 4]. There are sex differences in visuo-spatial abilities between males and females, for example, position perception, vision-for-action, and others [5]. Among these, visual motion perception is a crucial ability for understanding the dynamic external environment. This ability is believed to be neurally related to the function of the human MT complex (MT+, including MT and MST) [6, 7]. Therefore, we put forward a hypothesis that there are sex differences in the MT+ region, including structure and function. Investigating this hypothesis is the goal of our paper.

Using a well-known visual motion paradigm [8, 9], Murray et al. [10] reported that the motion duration thresholds were significantly shorter for males than females. Motion duration thresholds have been linked to motion processing within brain regions, particularly in MT+ [6, 8, 9, 11] area. Therefore, investigating sex differences in the structure and function of MT+ region is a necessary step in understanding the neural mechanisms. Many studies have investigated the sex differences of human brain using MRI technique. It has been consistently shown that sex differences were prominent in gray matter volume (GMV) of several specific brain regions, including occipital lobe [12–14]. The amplitude of low-frequency fluctuations (ALFF) reflects the intensity of spontaneous brain activity within a region [15, 16]. Our recent study [17] reported that ALFF was significantly related with visual motion processing in the MT+ region. In addition, previous findings [17–20] demonstrated that the MT+ region exhibits laterality in inducing perceptual effects. Together, these studies motivated us to examine sex differences in GMV and ALFF of the MT+ region, and to further compare the sex differences of these indexes between the left and right MT+ regions.

The amygdala is a subcortical structure with multiple subregions [21, 22], which shows sex differences in its activity [23–26]. Furthermore, there are many studies showing that the amygdala has bidirectional interactions with visual cortex [27–30]. We therefore also examined the sex differences in the inter-regional functional connectivity (FC) between MT+ and amygdala, as well as the FCs between MT+ and subregions of amygdala, including the laterobasal (LB), superficial (SF), and centromedial (CM).

Our work revealed that the faster motion perception for males compared to females, corroborating previous findings [10]. Using ultra-high field (UHF) MRI to investigate the structure and function of the MT+ region, we observed sex differences to be prominent in major brain parameters of left MT+ (rather than right MT+, i.e., laterality), including larger GMV and higher ALFF in left MT+, and stronger FC between the left MT+ and the left centromedial amygdala (CM) in males. Our findings also indicate that females and males share a similar neural mechanism underlying motion perception, characterized by larger GMV, higher ALFF in the left MT+ region, and shorter threshold of motion discrimination. Additionally, a mediation model demonstrates that the relationship between GMV of the left MT+ and duration threshold of small stimulus was indirect-only and mediated by ALFF of the left MT+. Together, our research shows sex differences in the structure and function of the MT+ region, which may be the neural mechanism underlying the sex difference in the temporal features of visual motion discrimination ability.

## Methods

### Subjects

There were two separate cohorts of participants (total *n* = 95) (Supplementary Table 1). Cohort 1 (*n* = 33) included 17 males and 16 females from 21 to 36 years age who participate in task-state fMRI experiment. Cohort 2 (*n* = 62) included 30 males and 32 females from 19 to 31 years age who participate in psychophysical experiments and structural MRI and resting-state fMRI experiments. All participants had normal or corrected-to-normal vision, and right-handedness. For all experiments, the sex of participants was determined as self-report biological male or female, thus the study focuses on biological sex differences. All procedures were approved by the Ethics Review Committee of Zhejiang University, and conducted in accordance with the Helsinki Declaration. All participants signed informed consent forms prior to the start of the study and were compensated for their time.

### Visual motion perception experiment

The visual stimuli were generated using Psychophysics Toolbox [31] on MATLAB (MathWorks, Natick, MA, USA), which displayed on a linearized monitor (1920 × 1080 resolution, 100 Hz refresh rate, Cambridge Research System, UK). During the experiment, a chin rest needed to stabilize head position, maintaining distance of 72 cm from the screen.

The details of the procedure for measurement are available in our recent study [11]. The stimulus used drifting sinusoidal luminance modulation gratings at 50% contrast. Motion speed was 4°/s, and spatial frequency was 1 cycle/°. The grating had two directions of motion (left and right) and two diameters (2° and 10°), thus combining to produce 4 different stimulus patterns. Stimuli were presented centrally on a gray (56 cd/m^2^) background, whose edges were blurred using a raised cosine function (width, 0.3°). The grating was ramped on and off with a Gaussian temporal envelope, and the grating duration was defined as 1 SD of the Gaussian function. The grating duration was adaptively adjusted on each trial and then estimated by a staircase procedure. Thresholds for large and small gratings were obtained from a 160-trial block that contained four interleaved three-down/one-up staircases. For each participant, we recorded the duration threshold and correctness rate for each of the two stimulus sizes. These values were then fitted to a cumulative Gaussian function, and the duration threshold corresponding to the 75% correct point on the psychometric function was estimated for each stimulus size. Stimulus demonstration and practice trials were presented before the first run. There is audio feedback for each incorrect response.

### MR experimental procedure

MR data were collected using a 7T whole body MR system (Siemens Healthcare, Erlangen, Germany) with a Nova Medical 32 channel array head coil. In the task-state fMRI of cohort 1, thirteen blocks were presented during a run (20 s each, 130TRs total). Each block contained 10 s of baseline and 10 s of stimulus presentation. The stimulus consisted of moving gratings (contrast = 98%, speed = 4°/s, diameter = 2°, spatial frequency = 3 cycle/°), which moved in one of eight possible directions in a randomized and count-balanced order for 400 ms, with an interval of 225 ms. Moreover, during the task, red dots randomly appeared in the center of the screen, and participants needed to press a key to respond in order to focus their attention. The functional magnetic resonance experiment system (SMARTEC, SA-9800) of Shenzhen Virtue Medical was used to present visual stimuli. Myopic subjects needed to wear magnetic resonance compatible glasses to ensure corrected visual acuity greater than 1.0.

In cohort 2, sessions included resting-state fMRI and structural MRI scanning. Resting-state scans were obtained with 1.5-mm isotropic resolution (transverse orientation, TR/TE = 2000/20.6ms, 160 volumes, slice number = 90, flip angle = 70°, eyes closed). Structural images were acquired using a MP2RAGE sequence (TR/TI1/TI2 = 5000/901/3200ms) with 0.7-mm isotropic resolution.

### MRI data processing and analysis

Task fMRI data were processed in AFNI [32]. The following steps were performed in order: motion correction, alignment to the T1 anatomy and detrend. MT+ ROI identified from the intersection of the two parts, one for the activation cluster, and the other for a sphere with a radius of 8 mm and the center point coordinates of the activation area. The activation cluster was obtained by false discovery rate (FDR) correction and then setting cluster size to 20. For each block, minimum signal amplitude in the first 3TRs was calculated as the response baseline, and the maximum signal amplitude in the last 3TRs was calculated as the response peak. Across blocks in each subject, the average of the percentage change from the response baseline to the response peak served as the signal change.

Resting-state functional images were preprocessed using the DPABI toolbox [33] based on Statistical Parametrical Mapping 12 (SPM12) including: removal of the first five volumes, slice timing, realignment, registration of anatomical and functional images for each subject, segmentation of the anatomical images through DARTEL, linear detrend, nuisance covariates regression (with realignment Friston 24-parameter, white matter and CSF signal, without global signal) [34], normalization to the standard Montreal Neurological Institute (MNI) space with a resolution of 1.5 × 1.5 × 1.5 mm^3^ using DARTEL, spatial smoothing with a 3 mm FWHM Gaussian filter, and band-pass filtering with frequency band of slow 3 (0.073–0.198 Hz), slow 4 (0.027–0.073 Hz) and slow 5 (0.01–0.027 Hz) [35, 36]. Data was included if the subject’s head movement during fMRI scanning was less than 3 mm translation.

CAT12 toolbox (https://www.nitrc.org/projects/cat/) is a free extension to SPM12 to provide computational anatomy. T1-images were preprocessed using default settings (https://neuro-jena.github.io/cat12-help/). The preprocessing procedures were as follows: first, 3D T1-weighted images are interpolated, normalized using an affine followed by non-linear registration, denoised, corrected for bias field inhomogeneities. Then, all corrected images were segmented into GM, WM, and cerebrospinal fluid (CSF) and normalized into the standard MNI space using DARTEL. The resulting images were checked for homogeneity. Finally, GM images were smoothened using an 8 mm FWHM Gaussian kernel.

Before GMV and ALFF analysis, we defined the region of interests (ROIs). The left MT+ and right MT+ were each defined by a sphere with a radius of 8 mm (centered with (− 43, − 73, − 4), (50, − 66, − 1) in MNI space, respectively). We took the left and right MT+ as well as amygdala subregions as ROIs and correlated their time series with each other (i.e., FC). Before calculating the FC, we defined left and right amygdala subregions of CM, LB, SF by a 50% probabilistic map, which was created by Amunts et al. [21].

### Statistical analysis

SPSS (version 20.0) was used to perform all statistical analysis in this study. We used student’s t test (mean ± SEM) to determine difference between males and females or within-group differences. Pearson’s correlation coefficient was used to measure the relationship between different variables (duration threshold, GMV and ALFF). Differences or correlations were considered statistically significant if *P* < 0.05. Significances with multiple comparisons were tested with FDR correction.

PROCESS version 3.4, a toolbox in SPSS, was used to examine the mediation model [37]. In this model, we treated the duration threshold as the outcome variable and GMV of MT+ as the predictor variable, with ALFF in MT+ as the mediator variable. We examined whether the relationship between MT+ GMV and duration threshold was mediated by ALFF in MT+. We reported 95% CIs based on 5000 bootstrap iterations for all major effects.

## Results

We collected task-fMRI data (drifting grating stimuli) in cohort 1, collected psychophysical data (measurement of motion perception) and multimodal MRI data (structural MRI and resting-state fMRI) in cohort 2 (“Methods” and Table S1). Motion perception has been linked to motion processing in the brain region of MT+, so in this study, we mainly examined the multimodal imaging indexes in the MT+.

### Sex differences in signal change of task-fMRI and visual motion perception

**Fig. 1:**
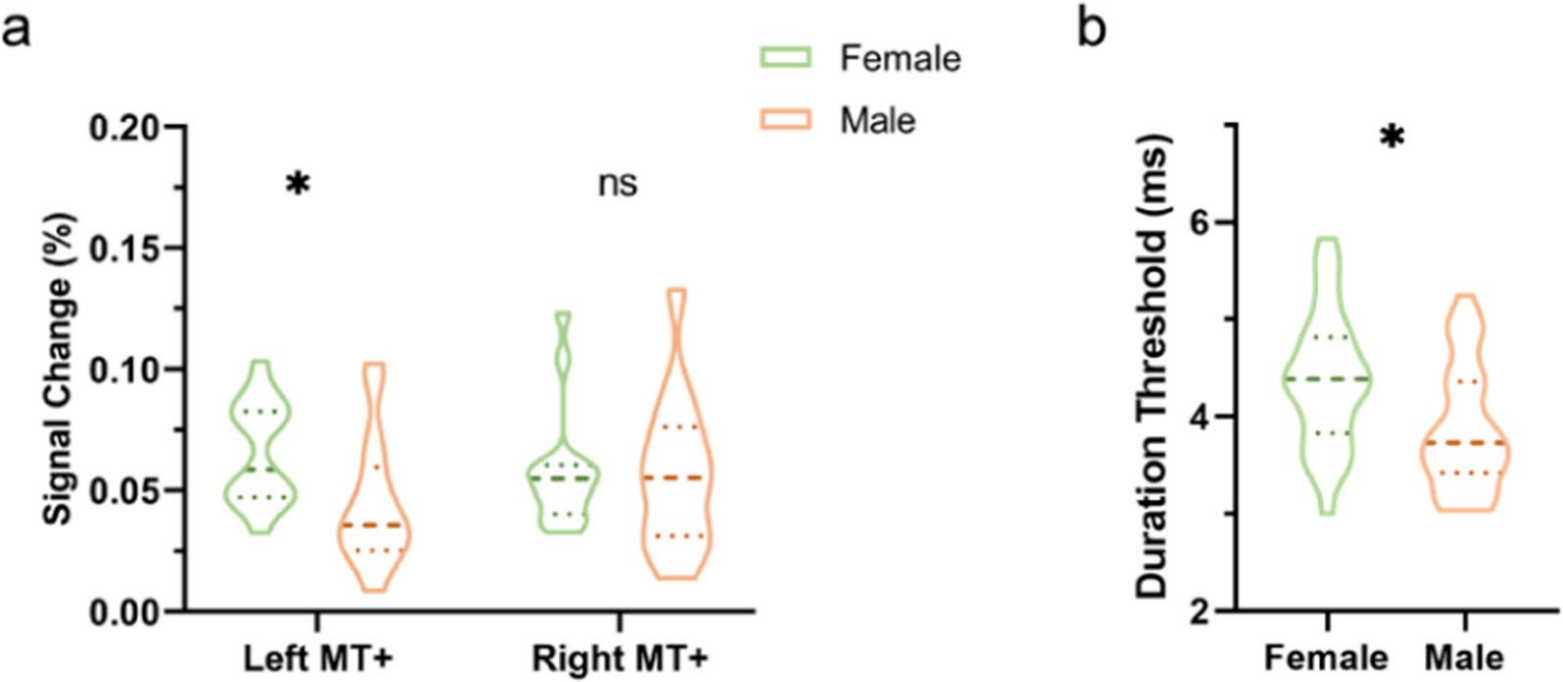
Sex differences in MT+ signal change percentage (cohort 1) and motion perception (cohort 2). **a** In cohort 1, signal change percentage of left MT+ in females (0.07 ± 0.01, *n* = 15) was significantly higher (*P*_*fdr*_ = 0.04) than in males (0.04 ± 0.01, *n* = 16), but there were no significant differences (*P*_*fdr*_ = 0.61) in signal change of right MT+ between males (0.05 ± 0.01, *n* = 16) and females (0.06 ± 0.01, *n* = 15). **b** In cohort 2, males (3.88 ± 0.12 ms, *n* = 30) had significantly (*P* = 0.01) shorter duration thresholds of small stimulus than females (4.37 ± 0.12 ms, *n* = 31). The thick dashed line represents the median and the thin dashed line represents the quartiles. Two-sample *t*-test, significance threshold ∗*P*_*fdr*_ < 0.05 and ns: no significance, all *P* values had been corrected (FDR)

In cohort 1, we measured the fMRI blood oxygen level-dependent (BOLD) signal change to drifting grating stimuli relative to blank fixation condition, which directly reflects the neural response of the MT+ region to moving stimuli. Surprisingly, we found that the signal change percentage of left MT+ was significantly (*P*_*fdr*_ = 0.04) higher in females (0.07 ± 0.01, *n* = 15) than in males (0.04 ± 0.01, *n* = 16), while there were no significant (*P*_*fdr*_ = 0.61) sex differences in the signal change percentage of the right MT+ (Fig. 1a). In addition, there were no significant sex differences in the signal change percentage of bilateral MT+ (Fig. S1), which was consistent with a previous study [10].

In Cohort 2, we measured duration thresholds using a well-known visual motion paradigm, which refers to the minimum durations needed to accurately perceive motion direction [8, 9]. We found significant differences (*P* = 0.01) in the durations of small stimulus threshold (ST) between females (4.37 ± 0.12 ms, *n* = 31) and males (3.88 ± 0.12 ms, *n* = 30) (Fig. 1b), whereas there were no significant differences (*P* = 0.29) in the duration thresholds of large stimulus between females (5.22 ± 0.22 ms, *n* = 31) and males (4.94 ± 0.12 ms, *n* = 30) (Fig. S2). These results on the small stimulus was consistent with a previous study across three laboratories and a total of 263 participants [10]. We did not find the similar sex differences in the duration threshold of the large stimulus as in the previous study [10], which may be due to the difference in the way of blurred edges of grating stimuli (i.e., Gaussian blurring in the study of Murray et al. [10], but raised cosine blurring in our study). Next, we analyzed the multimodal imaging data using 7T MRI scanning in cohort 2 subjects.

### Sex differences in multimodal MRI indexes

**Fig. 2:**
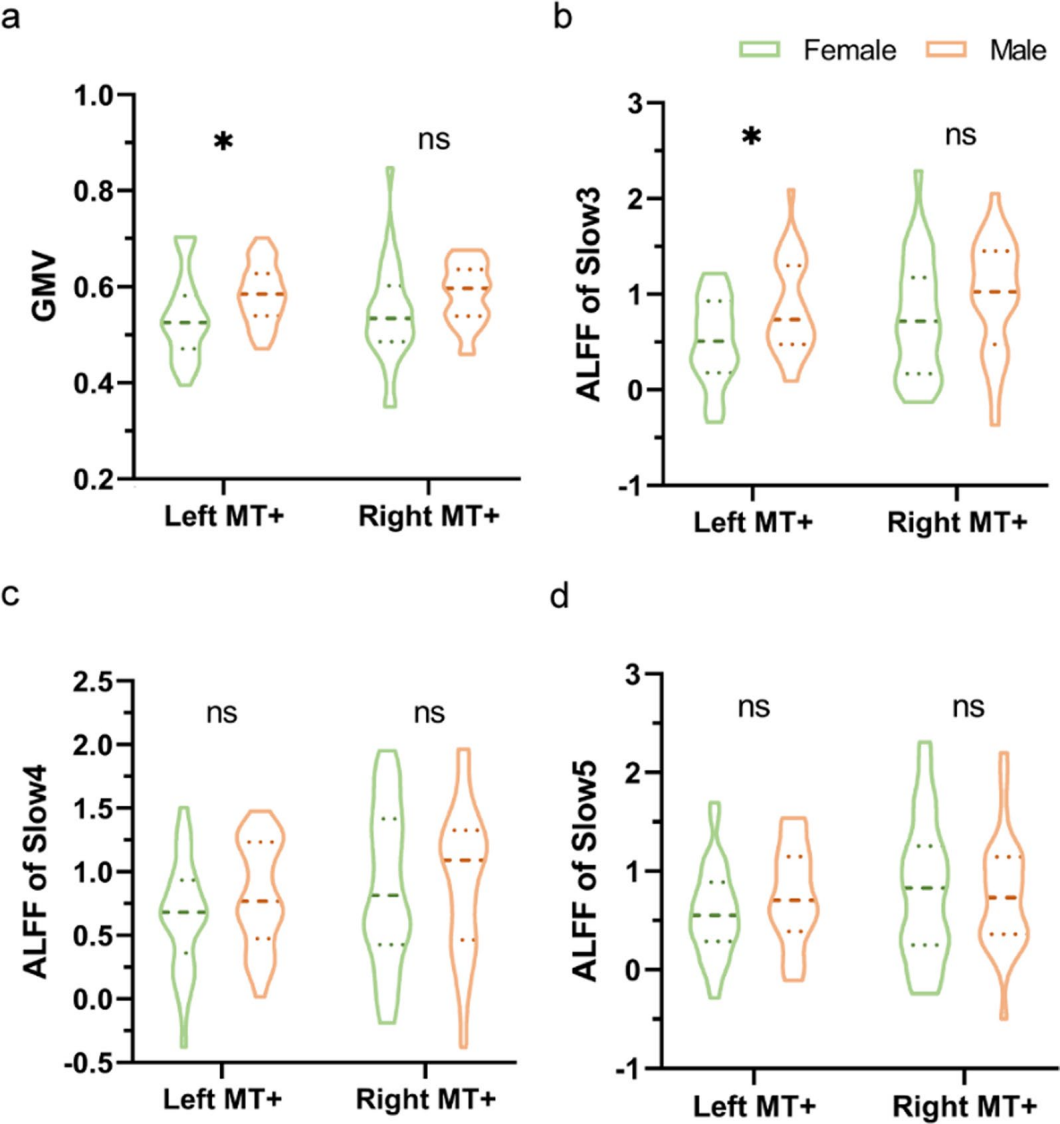
Sex differences in structural and functional imaging values of MT+. **a** The GMV values of left MT+ were significantly larger (*P*_*fdr*_ = 0.04) in males (0.59 ± 0.01, *n* = 28) than females (0.54 ± 0.01, *n* = 32). There were no significant differences (*P*_*fdr*_ = 0.06) of GMV values in right MT+ between males and females. **b** The ALFF values of left MT+ in slow 3 frequency (0.073–0.198 Hz) were significantly higher (*P*_*fdr*_ = 0.01) in males (0.87 ± 0.09, *n* = 28) than females (0.51 ± 0.08, *n* = 31). While there were no significant differences (*P*_*fdr*_ = 0.13) in ALFF slow 3 values in right MT+ between males (1.06 ± 0.10, *n* = 28) and females (0.76 ± 0.11, *n* = 31). **c** There were no significant differences of ALFF values in slow 4 frequency band (0.027–0.198 Hz) between males and females in both left and right MT+ regions. **d** There were also no significant differences of ALFF values in slow 5 frequency band (0.01–0.227 Hz) between males and females in both left and right MT+ regions

The laterality of sex differences in signal change percentage let us examine the multimodal MRI parameters of left and right MT+ respectively in our subjects. To investigate whether there were sex differences in left or right MT+ of structural MRI and resting-state fMRI indexes, we calculated the values of GMV and ALFF of the BOLD signal in cohort 2. According to our analysis, the GMV values of left MT+ were significantly larger (*P*_*fdr*_ = 0.04) in males (0.59 ± 0.01, *n* = 28) than in females (0.54 ± 0.01, *n* = 32), while the GMV values of right MT+ showed no significant sex differences (*P*_*fdr*_ = 0.06) (Fig. 2a). In ALFF analysis, we subdivided the infra-slow frequency range into its subcomponents: slow 5 (0.01–0.027 Hz), slow 4 (0.027–0.073 Hz) and slow 3 (0.073–0.0198 Hz) (“Methods”). This yielded significant (*P*_*fdr*_ = 0.01) higher ALFF values in left MT+ of males (0.86 ± 0.09, *n* = 30) compared to females (0.51 ± 0.08, *n* = 31) in the fastest infra-slow frequency of slow 3. There was no significant sex difference (*P*_*fdr*_ = 0.06) in right MT+ for the ALFF values in slow 3 frequency band (Fig. 2b). For the slower infra-slow frequency bands of slow 4 and slow 5, there were no significant sex differences of ALFF values both in left and right MT+ regions (Fig. 2c and d).

**Fig. 3:**
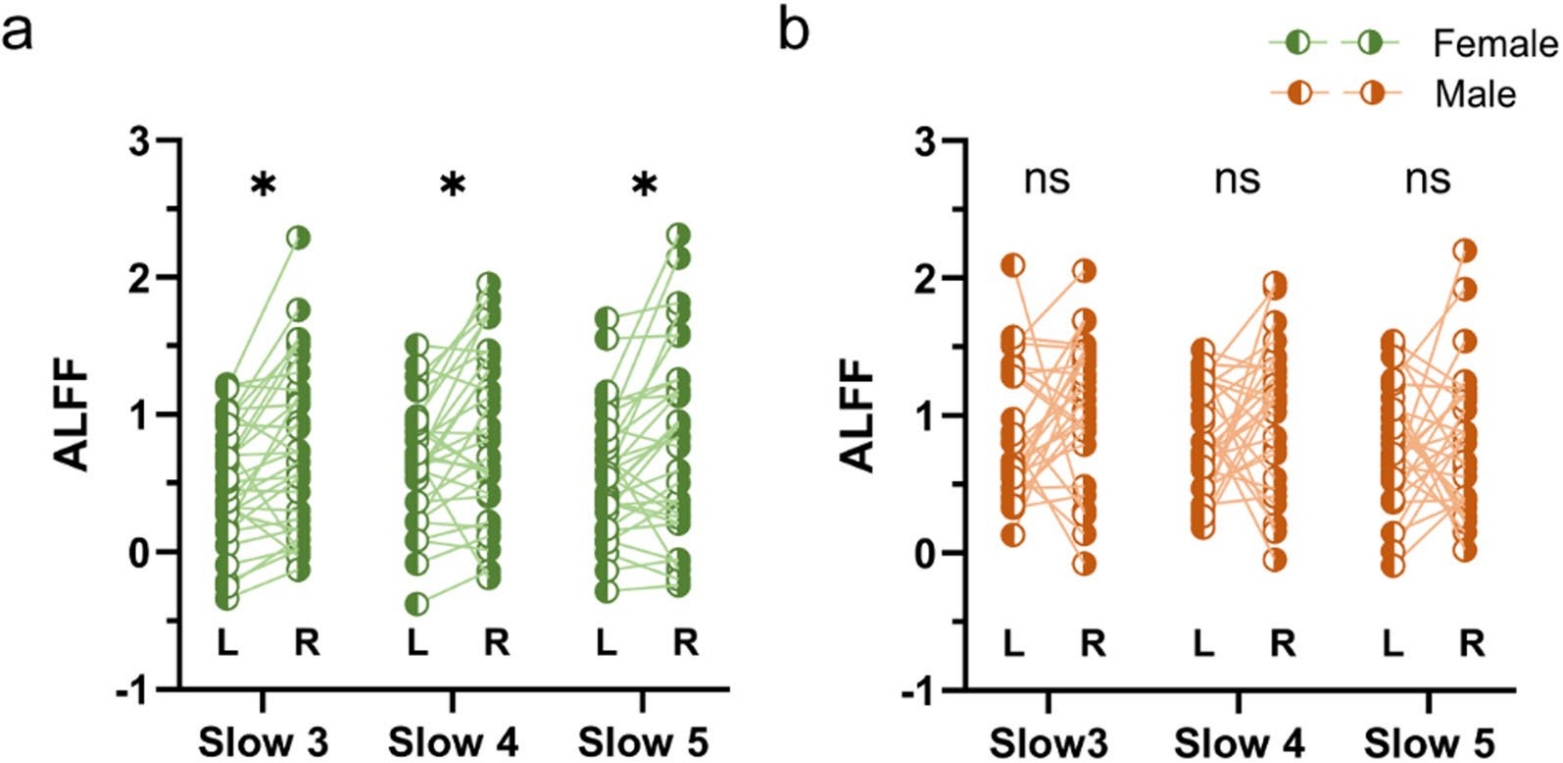
ALFF differences between left and right MT+ regions in intragroup. **a** In females, there were significantly higher ALFF values in right MT+ region compared to left MT+ region in all of the three infra-slow frequency bands, slow 3 (*P*_*fdr*_ = 0.01), slow 4 (*P*_*fdr*_ = 0.01), and slow 5 (*P*_*fdr*_ = 0.01). **b** In males, there were no significant differences of ALFF values between left and right MT+ regions in all of the three infra-slow frequencies, slow 3 (*P*_*fdr*_ = 0.49), slow 4 (*P*_*fdr*_ = 0.28), and slow 5 (*P*_*fdr*_ = 0.67)

In comparison of ALFF differences between left and right MT+ regions within cohort 2, we found that females had significantly higher ALFF values in right MT+ than left MT+ in all of the three infra-slow frequency bands (slow 3, slow 4 and slow 5, Fig. 3a). While males showed almost no differences between left and right MT+ ALFF in all of the three infra-slow frequency bands (slow 3, slow 4 and slow 5, Fig. 3b).

### *MT* + *GMV and ALFF values relate to duration thresholds and mediation modal*

A recent study [10] found that individual differences in psychophysical thresholds correlated with individual differences in the signal change percentage of MT+ fMRI. This inspired us to explore if the MT+ structural and resting-fMRI indexes (e.g., GMV and ALFF values) can predict the inter-individual differences of the duration thresholds.

**Fig. 4:**
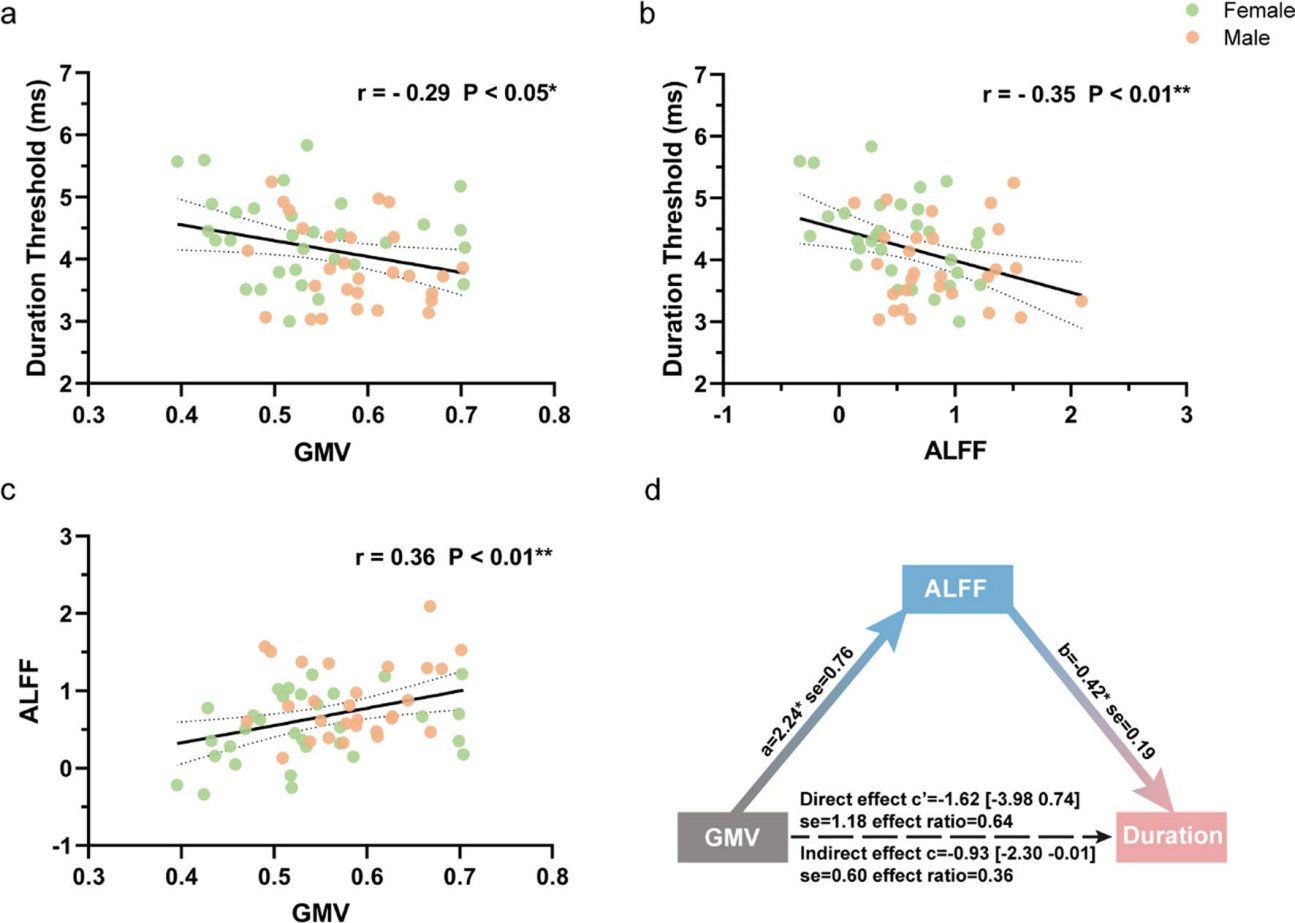
Correlations among inter-individual’s variables of left MT+ GMV, ALFF in slow 3 and ST and mediation model. **a** Significantly negative correlation between duration thresholds of small stimuli (ST values) and the GMV values of left MT+. **b** Significantly negative correlation between ST values and the ALFF values in slow 3 frequency band within left MT+. **c** Significantly positive correlation between left MT+ GMV and ALFF slow 3 values. **d** Mediation analysis among the three variables of ST, left MT+ GMV and ALFF in slow 3, which yields that the relationship between left MT+ GMV and ST values was indirect-only and mediated by ALFF in slow 3 in left MT+

We found that the duration thresholds of small stimulus (ST) significantly negatively correlated with both left MT+ GMV (*r* = − 0.29, *P* = 0.03, Fig. 4a) and left MT+ ALFF in slow 3 frequency band (*r* = − 0.35, *P* = 0.01, Fig. 4b). Furthermore, the GMV values of left MT+ significantly positively correlated with the ALFF values in slow 3 frequency in left MT+ (*r* = 0.36, *P* = 0.01, Fig. 4c). ST values also significantly negatively correlated with both right MT+ GMV (*r* = − 0.28, *P* = 0.03) and right MT+ ALFF in slow 3 (*r* = − 0.28, *P* = 0.03). There was no significant correlation between right MT+ GMV and right MT+ ALFF in slow 3 (*r* = 0.19, *P* = 0.15). Separating the male and female groups, aside from a significant correlation between left MT+ ALFF in slow 3 and ST in the female group, there were no significant pairwise correlations among left MT+ GMV, left MT+ ALFF in slow 3, and ST within either the male or female groups (Fig. S3).

In order to more fully investigate the relationship among psychophysical thresholds (ST values), GMV values and ALFF in slow 3 values in left MT+, we applied a mediation analysis [37]. Mediation approaches assess the mechanism through which two variables are related. Mediation is well suited when the mediating variable (Mi) correlates with both independent (X) and dependent (Y) variable; it can be used to show whether Mi mediates the effect of the independent upon the dependent variable. We tried the mediation model in cohort 2 subjects. The analysis yielded a significant indirect effect, where the ALFF in slow 3 in left MT+ (Mi) mediated the relationship between left MT+ GMV (X) and ST values (Y) (Indirect effect: c = − 0.93, [BootLLCI, BootULCI] = [− 2.30, − 0.01]; se = 0.60; effect ratio = 0.36), whereas there was no significant direct effect of X and Y variable (Direct effect: c’ = − 1.62, [LLCI, ULCI] = [− 3.98 0.74]; se = 1.18; effect ratio = 0.64). This suggests that, neuronal activity in the fastest infra-slow frequency band in left MT+ (ALFF in slow 3) mediates the relationship of left MT+ structure (GMV) and motion discrimination thresholds (ST) (Fig. 4d).

### *Sex differences in FCs between MT*+* and amygdala subregions*

**Fig. 5:**
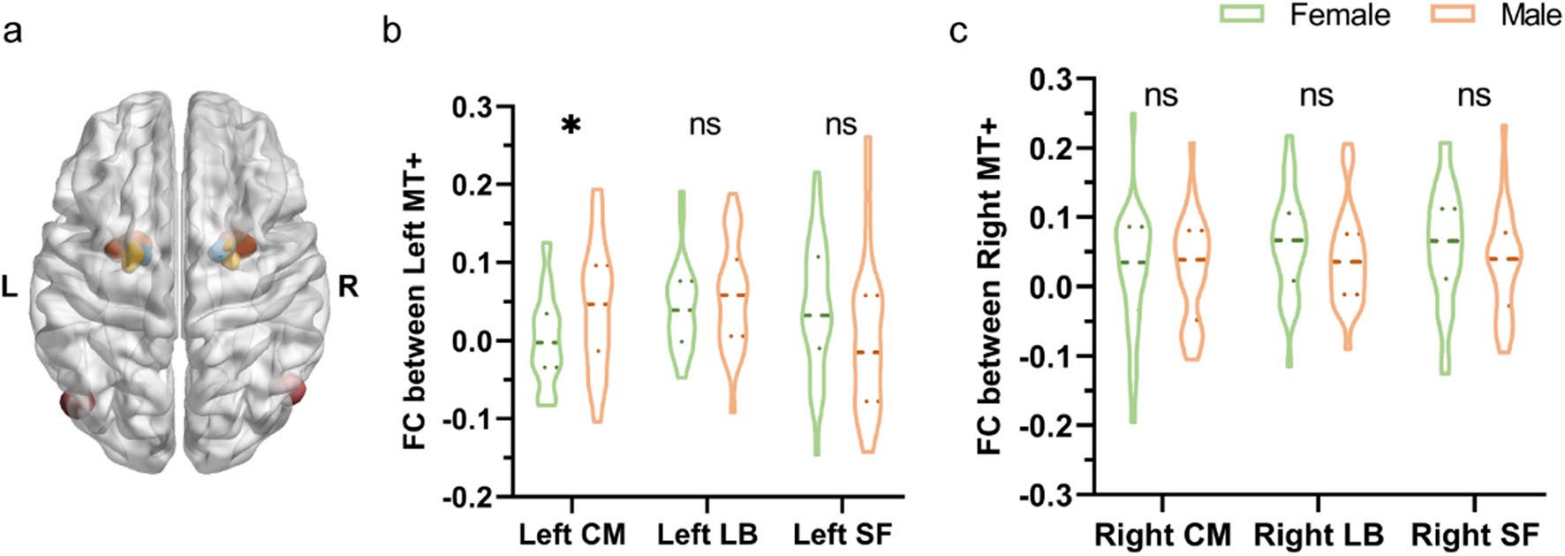
Sex differences in FCs between MT+ and ipsilateral amygdala subregions. **a** Yellow color signifies the CM subregion of amygdala, orange color indicates the LB subregion of amygdala, blue color stands for the SF subregion of amygdala, and pink color relates to the MT+ region. **b** There were significantly higher (*P* = 0.02) FC values of left MT+ and left CM subregion of amygdala in males (0.04 ± 0.01, *n* = 30) compared to females (0.00 ± 0.01, *n* = 30), while there were no significant sex differences of FC values between left MT+ and left LB and SF subregions of amygdala. **c** There were no significant sex differences of FC values between right MT+ and right CM, LB and SF subregions of amygdala

A body of research have revealed sex differences in amygdala activity [23–26], and many studies reported that the amygdala has bidirectional interactions with visual cortex [27–30]. We thus raised two questions: (1) whether there were sex differences in FC between MT+ and amygdala; (2) whether there was laterality of sex differences. Firstly, we took left and right MT+ as well as left and right amygdala as regions of interest (ROI) and correlated their time series with each other (i.e., FC). There were no significant sex differences between males and females in the FCs of left MT+ and left amygdala (*P* = 0.57), left MT+ and right amygdala (*P* = 0.73), right MT+ and right amygdala (*P* = 0.29), and right MT+ and left amygdala (*P* = 0.80) (Fig. S4a and b). The amygdala can be anatomically divided into three nuclei (Fig. 5a): the laterobasal (LB), superficial (SF), and centromedial (CM), each of which features distinct functions [38]. Therefore, we did FC analyses between left/right MT+ and left/right amygdala subregions (LB, SF and CM). We found that the FCs between left MT+ and left CM were significantly stronger (*P* = 0.02) for males (0.04 ± 0.01, *n* = 30) than females (0.00 ± 0.01, *n* = 30) (Fig. 5b). There were no significant sex differences in the FCs of left MT+ and left LB/SF (Fig. 5b), and right MT+ and right CM/LB/SF (Fig. 5c). Furthermore, we also found no significant sex differences in the FCs of left MT+ and right CM/ LB/SF (Fig. S5a), and right MT + and left CM/LB/SF (Fig. S5b). Together, we revealed significant sex differences in FC between MT+ and CM subregion of amygdala, with laterality towards the left hemisphere in the differences.

## Discussion

In the present study, we re-examined sex differences in visual motion perception and investigated their neural basis using both structural and functional MRI data. We found significantly shorter duration thresholds in males compared to females, which is in line with a previous study [10]. To identify the potential neural mechanisms, we employed UHF MRI to demonstrate sex differences focusing on the structural and functional features of visual cortical area MT+. This included larger GMV (Fig. 3a) and higher ALFF in slow 3 in left MT+ (Fig. 3b) in males.

Overwhelmingly, behavior is similar in males and females, which supports the gender similarity hypotheses [39]. There are several apparent exceptions to the overall trend toward similar performance between males and females [40], though. For example, the reported sex differences in visual motion perception in this work and a previous study [10]. Furthermore, our results show that the sex differences in imaging data were of degree, but not of kind (Figs. 1, 2, 4 and 5), which is consistent with recently documented research [4, 41, 42]. Additionally, our findings in the brain-behavior correlations highlight a similar neural mechanism underlying the motion perception for both females and males. Specifically, this involves larger GMV, higher ALFF in slow 3 in left MT+, and shorter motion discrimination of small stimuli (Fig. 4a, b).

Previous studies have reported that males tend to have a larger visual cortex than females, including primary visual cortex and motion processing cortex (MT+) [14, 43, 44]. These researchers speculated that sex differences in brain size might predict the sex differences in visual perception [45]. In this work, we further identified the left MT+ as the key region that showed a significant difference between males and females. Indeed, numerous documented sex differences, such as those related to language or empathy, prominently exhibit lateralization in the left hemisphere [41, 45, 46]. In this study, the sex differences in visual motion processing are reflected in the brain imaging indexes of the left MT+, from structure (GMV) to resting-state function (ALFF) and task-state function (signal change percentage).

In cohort 1, signal change percentage in the left MT+ in females was significantly higher than in males, while no significant sex difference was observed in the signal response to drifting sinewave gratings in the right MT+ (Fig. 1a). In a previous study, Murray et al. [10] found no significant sex difference in fMRI response amplitude in the bilateral MT+. In line with that study, the sex differences disappeared when we also calculated signal change percentage using bilateral MT+ activated regions (Fig. S1). In cohort 2, we found significantly shorter duration threshold of small stimulus for males than females (Fig. 1b), which again is line with previous research [10]. One possible explanation for this sex difference is the “hunter-gatherer theory” [1, 47, 48], it is necessary for males to be more sensitive in perceiving motion when engaging in hunting activities far from their residential areas. Previous studies have indicated that females exhibit superior verbally mediated memory and emotional memory [42, 43], which further support the potential evolutionary causes of sex differences.

In the present study, we also explored neural features (e.g., GMV and ALFF values) underlying the sex differences, which may contribute to the differences of behavioral performance (ST values) between males and females. Our results showed that there were significantly larger GMV in left MT+ for males than females (Fig. 2a). Neural spontaneous activity (e.g., ALFF values) in left MT+ was significantly higher in males compared to females in the fastest infra-slow frequency band of slow 3 (0.073–0.198 Hz) (Fig. 2b). While there were no significant sex differences in slower infra-slow frequency bands of slow 4 (0.027–0.0073 Hz, Fig. 2c) and slow 5 (0.01–0.027 Hz, Fig. 2d). Furthermore, we tried a mediation model among the ST values, left MT+ GMV values and ALFF values in slow 3. We found that the relationship between left MT+ GMV and ST values was indirect-only and mediated by ALFF slow 3 in left MT+ (Fig. 4d). Together, these results support for the male dominance bias in motion perception, that is, from motion processing cortex (MT+) to behavior performance.

**Fig. 6:**
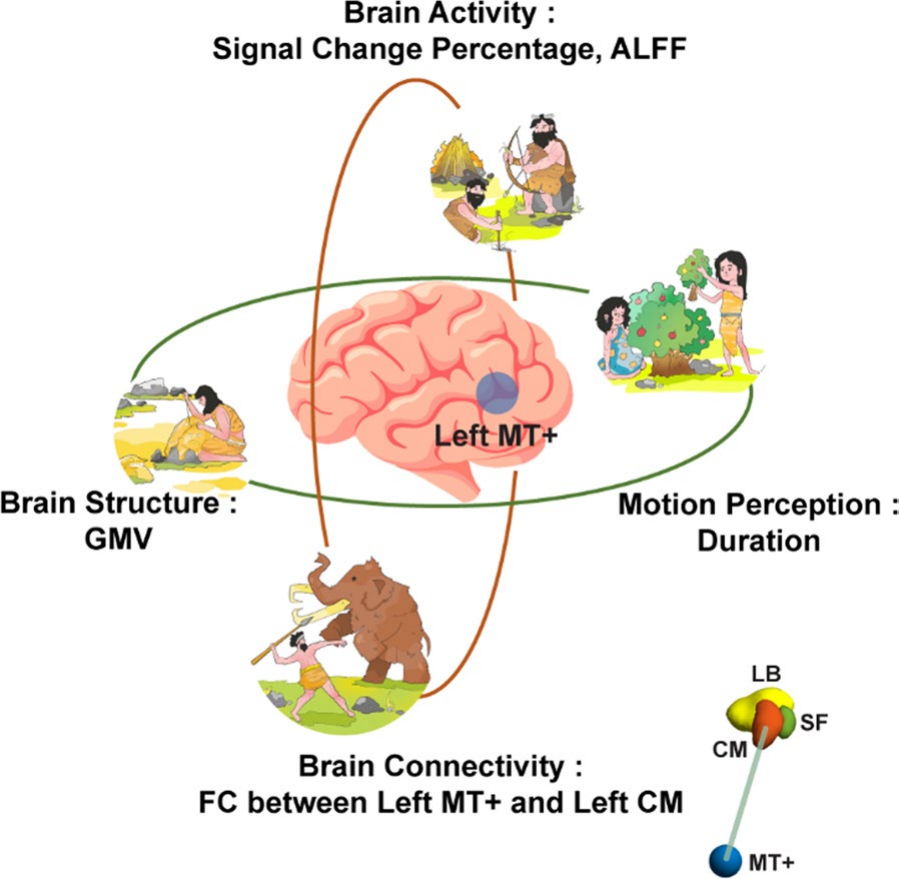
Cartoon displays one interpretation of this study. The results in our study might be consistent with different roles of females and males as laid out in the “hunter-gatherer theory”

In addition, we showed significant sex differences in FCs between the left MT+ and the left CM of amygdala, which were stronger in males than females (Fig. 5b). The CM is believed to be an important output region [22], and is also reported to modulate attentional allocation, cortical vigilance, and predatory hunting [22, 49, 50]. The LB is the main nucleus that receives sensory inputs [51], primarily engaged in the updating and evaluation of sensory stimuli [22, 51]. The SF subregion involves in olfactory and olfactory-related processing [22, 52]. Therefore, the significant sex differences of FCs between MT+ and CM indicate that the synchronous activity between MT+ and CM is notably stronger in males than in females. Furthermore, we revealed significant sex differences of FC via ipsilateral connections (left MT+ and left CM). This supports previous findings that the amygdala exhibits direct projections to many ipsilateral areas along the cortical visual pathways [27, 52–54]. From an evolutionary perspective, ipsilateral projection, e.g., not through the corpus callosum, allows for quicker processing of survival-related information, such as threats from predators [55]. These results can also be explained by the “hunter-gatherer theory” [1, 47, 48], Fig. 6 displays that the sex differences in this study might relate to different roles of males and females in early labor division, i.e., hunters or gatherers.

Our research and other relevant studies indicate the existence of sex differences in the human brain [41, 42]. Mental and psychological disorders show sex differences. For example, epidemiological surveys reveal a higher prevalence of psychological issues among females compared to males. Further, men may reduce help-seeking behaviors when encountering such problems [56–58]. Research on sex differences in the brains of healthy participants can contribute to identifying pathological pathways and treatment targets for personalized diagnosis and treatment of disorders across sex [59]. Our study highlights sex differences in behavior and brain function related to MT+. Considering the functional abnormalities in MT+ among MDD patients [11, 17, 60] and the role of MT+ in cognition [61], this suggests sex differences in the pathophysiological mechanisms of MDD related to MT+.

There are several limitations that should be considered. Firstly, we did not discuss the influence of gene and environment on the sex differences in the human brain. Such factors undeniably impact the structure and function of the brain [56, 62], including brain regions involved in motion perception [63]. Secondly, previous studies have reported that multiple psychiatric disorders exhibit the abnormality of motion perception. For instance, enhanced performance has been observed in the participants with autism spectrum disorder [64], while significantly longer motion discrimination thresholds were noted in schizophrenia and depression patients [11, 65]. In this study, we did not explore the role of motion perception in its disease-related aspects. Future research should rigorously examine sex differences in the abnormal features of these diseases [66], including visual motion perception, as well as the structure and function of related brain regions.

In conclusion, the present study highlights significant sex differences in the structure and function of the left MT+ region. Further, brain-behavior correlation analyses show a significant relationship of the duration threshold of motion discrimination with the imaging indexes of the left MT+ region, e.g., the values of GMV and ALFF in slow 3. These results supported that behavioral sex differences in visual motion perception are mainly related to the structure and function of the MT+ region.

### Perspectives and significance

Our findings suggest that sex differences in visual motion perception are related to sex differences in its functional brain regions (e.g. MT+), which highlights the importance of sex as a factor in both human brain and behavioral research.

## Supplementary Information

Supplementary Material 1.

## Data Availability

All data needed to evaluate the conclusions in the paper are present in the paper and/or the Supplementary information. Datasets are available from the corresponding authors on reasonable request.

## Code availability

This paper does not report original code.
