# Supplementary material for: Sex differences in the human brain related to visual motion perception
Source: Biol Sex Differ. 2024 Nov 11;15:92. doi: 10.1186/s13293-024-00668-2 (PMC11552312; doi:10.1186/s13293-024-00668-2)
Supplement: Supplementary file 1 — Supplementary Material 1. [file 13293_2024_668_MOESM1_ESM.docx]

**Supplementary Information**

**Sex differences in the human brain related to visual motion perception**

Dong-Yu Liu^1#^, Ming Li^2#^, Juan Yu^3^, Yuan Gao^1^, Xiaotong Zhang^4, 5^, Dewen Hu^2^, Georg Northoff^6^, Xue Mei Song^1, 3, 4 *^, Junming Zhu^1, 4*^

*Correspondence: [dr.zhujunming@zju.edu.cn](mailto:dr.zhujunming@zju.edu.cn), [songxuemei@zju.edu.cn](mailto:songxuemei@zju.edu.cn).

**This PDF file includes:**

Figures S1 to S5

Table S1

**Supplementary Figures and Legends**

**
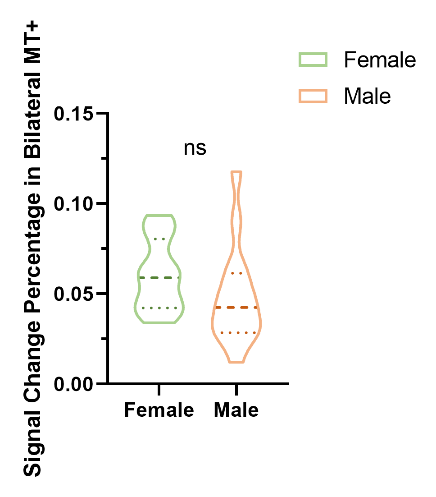
**

**Fig. S1 No sex differences in the signal change percentage of bilateral MT+.**

There were no significant sex differences (*P* = 0.21) in the signal change percentage of bilateral MT+ for males (0.06 ± 0.01, *n* = 14) and females (0.05 ± 0.01, *n* = 14).


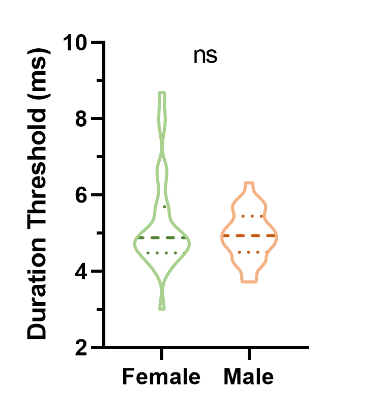


**Fig. S2 No sex differences in the duration thresholds of large stimulus.**

There were no significant differences (*P* = 0.29) of duration thresholds in large stimulus for males (4.94 ± 0.12ms, *n* = 30) compared to females (5.22 ± 0.22ms, *n* = 31). The thick dashed line represents the median and the thin dashed line represents the quartiles. Two-sample 𝑡-test, ns: no significance.

**
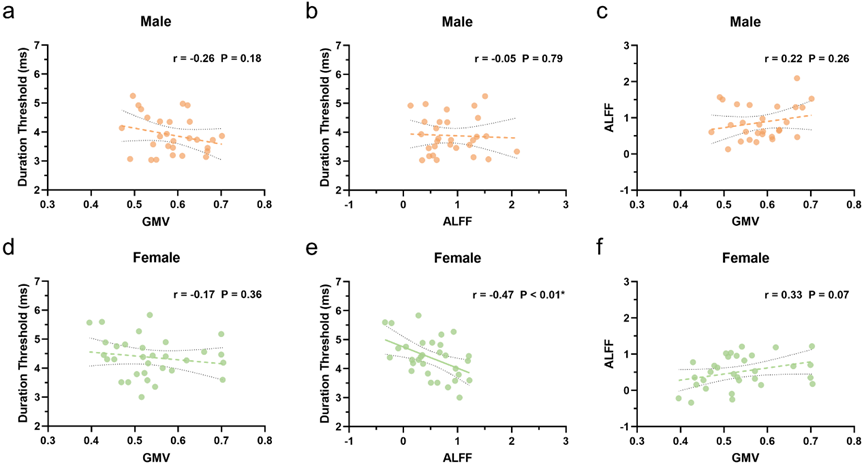
**

**Fig. S3 Correlations among inter-individual’s variables of left MT+ GMV, ALFF in slow 3 and ST within the male and female groups.**

**(a)** No significant correlation between duration thresholds of small stimuli (ST values) and the GMV values of left MT+ in the male group. (**b)** No significant correlation between ST values and the ALFF values in slow 3 frequency band in left MT+ in the male group. (**c)** No significant correlation between left MT+ GMV and ALFF slow 3 values in the male group. **(d)** No significant correlation between duration thresholds of small stimuli (ST values) and the GMV values of left MT+ in the female group. (**e)** A significant negative correlation between ST values and the ALFF values in slow 3 frequency band in left MT+ in the female group. **(f)** No significant correlation between left MT+ GMV and ALFF slow 3 values in the female group.

**
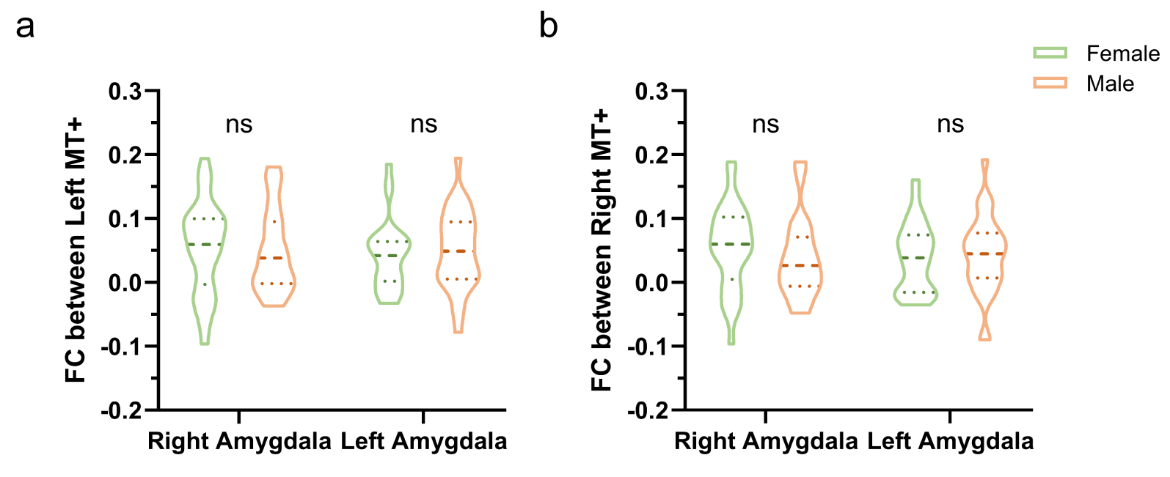
**

**Fig. S4** No significant of sex differences in FCs of MT+ and amygdala.

**(a)** There were no significant differences in FC values of left MT+ and right amygdala (*P* = 0.73) and FC values of left MT+ and left amygdala (*P* = 0.57). **(b)** There were no significant differences in FC values of right MT+ and right amygdala (*P* = 0.29) and FC values of right MT+ and left amygdala (*P* = 0.80).

**
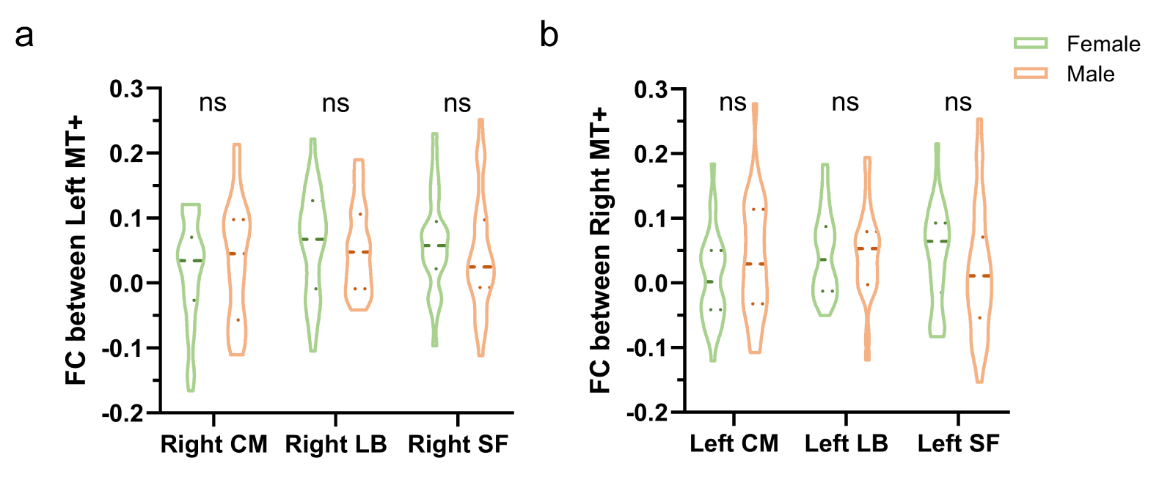
**

**Fig. S5** No significant of sex differences in FCs of MT+ and contralateral amygdala subregions

1. There were no significant differences in FC values of left MT+ and right CM subregion of amygdala (*P* = 0.44), FC values of left MT+ and right LB subregion of amygdala (*P* = 0.62), and FC values of left MT+ and right SF subregion of amygdala (*P* = 0.39). **(b)** There were no significant differences in FC values of right MT+ and left CM subregion of amygdala (*P* = 0.11), FC values of right MT+ and left LB subregion of amygdala (*P* = 0.86), and FC values of right MT+ and left SF subregion of amygdala (*P* = 0.27).

**Table S1. Descriptive statistics (Mean ± SD) of demographic information**

|  |  | Male |  | Female |  | *P* value |
| --- | --- | --- | --- | --- | --- | --- |
| Cohort 1 |  | N=17 |  | N=16 |  | – |
| Age (years) |  | 25.00±2.35 |  | 23.00±2.15 |  | 0.02 |
| Education (years) |  | 17.24±2.86 |  | 16.81±1.84 |  | 0.73 |
|  |  |  |  |  |  |  |
| Cohort 2 |  | N=30 |  | N=32 |  | – |
| Age (years) |  | 23.07±2.06 |  | 23.25±2.46 |  | 0.76 |
| Education (years) |  | 17.07±2.06 |  | 17.25±2.46 |  | 0.76 |

Differences in age and education were analyzed using two-sample t-test.
